# Impact of device scaling on the electrical properties of MoS_2_ field-effect transistors

**DOI:** 10.1038/s41598-021-85968-y

**Published:** 2021-03-23

**Authors:** Goutham Arutchelvan, Quentin Smets, Devin Verreck, Zubair Ahmed, Abhinav Gaur, Surajit Sutar, Julien Jussot, Benjamin Groven, Marc Heyns, Dennis Lin, Inge Asselberghs, Iuliana Radu

**Affiliations:** 1 IMEC, Leuven, Belgium; 2 KU Leuven, Leuven, Belgium

**Keywords:** Nanoscale materials, Nanoscience and technology, Nanoscale devices, Electronic devices

## Abstract

Two-dimensional semiconducting materials are considered as ideal candidates for ultimate device scaling. However, a systematic study on the performance and variability impact of scaling the different device dimensions is still lacking. Here we investigate the scaling behavior across 1300 devices fabricated on large-area grown MoS_2_ material with channel length down to 30 nm, contact length down to 13 nm and capacitive effective oxide thickness (CET) down to 1.9 nm. These devices show best-in-class performance with transconductance of 185 μS/μm and a minimum subthreshold swing (SS) of 86 mV/dec. We find that scaling the top-contact length has no impact on the contact resistance and electrostatics of three monolayers MoS_2_ transistors, because edge injection is dominant. Further, we identify that SS degradation occurs at short channel length and can be mitigated by reducing the CET and lowering the Schottky barrier height. Finally, using a power performance area (PPA) analysis, we present a roadmap of material improvements to make 2D devices competitive with silicon gate-all-around devices.

## Introduction

CMOS technology has advertently followed Moore’s law of device scaling for the past 50 years to achieve higher transistor density, higher speed and power improvements. A significant part of this device scaling, especially for the planar Metal–Oxide–Semiconductor-Field-Effect-Transistor (MOSFET) was achieved by scaling the gate length^1^. This scaling is reaching its limits as short channel effects (SCE) significantly degrade the device performance. To partially overcome SCE, the tri-gate (FinFET) structure has been introduced^2^. For future technology nodes, the gate-all-around nanosheet FET, which sandwiches thin layers of silicon channel between multiple gates, is expected to provide additional improvements. Both configurations enhance the electrostatic control over the channel and allow for further gate length scaling. However, it has been reported^3^ that the required silicon channel thickness scaling below 10 nm severely degrades the carrier mobility due to increased surface-roughness scattering. In this context, two-dimensional (2D) semiconducting materials such as transition metal-dichalcogenides (TMDs) are considered to be ideal candidates due to their naturally passivated surface and ultra-thin body (1 monolayer MoS_2_ ~ 0.65 nm), providing excellent gate-control and enhanced transport^4–7^. However, since many studies are performed with manually exfoliated flakes and collecting large datasets is very labor-intensive, there has been a strong focus on only selecting top performing devices, at the cost of less device understanding. Until recently, only a few TMD studies^8–10^ have focused on devices fabricated using large area grown films. Especially for device scaling^11^, a statistically significant set of data is still lacking.

Therefore, we carry out a study of the impact of geometrical scaling on an extensive data set of large-area grown tri-layer MoS_2_ MOSFETs (1300 devices). We investigate the impact of scaling the channel length (L_ch_) and width (W_ch_), contact length (L_cont_) and effective oxide thickness (EOT) on various device performance metrics such as the on- and off-current (I_on_, I_off_), contact resistance (R_c_), subthreshold swing (SS), interface trap density (D_it_) and threshold voltage (V_T_). We demonstrate that scaling the contact length down to 13 nm has no impact on the device performance. This confirms that carrier injection occurs exclusively from the edge of the metal directly into the thin TMD channel, which is in line with our TCAD simulations. Further, using our large data set, we make a detailed assessment on the scaling trends of SS and V_T_ with device dimensions. We identify the variation in the number of MoS_2_ layers in the channel and contact regions as a possible source for SS degradation and V_T_ variability for ultra-scaled TMD MOSFETs. Such insights are crucial for device understanding and enables device architectures such as double-gate^12^ or stacked TMD FETs to outperform Si FETs^13^. This article is an extension of our previous work presented at IEDM 2019^10^.

## Results and discussion

**Figure 1:**
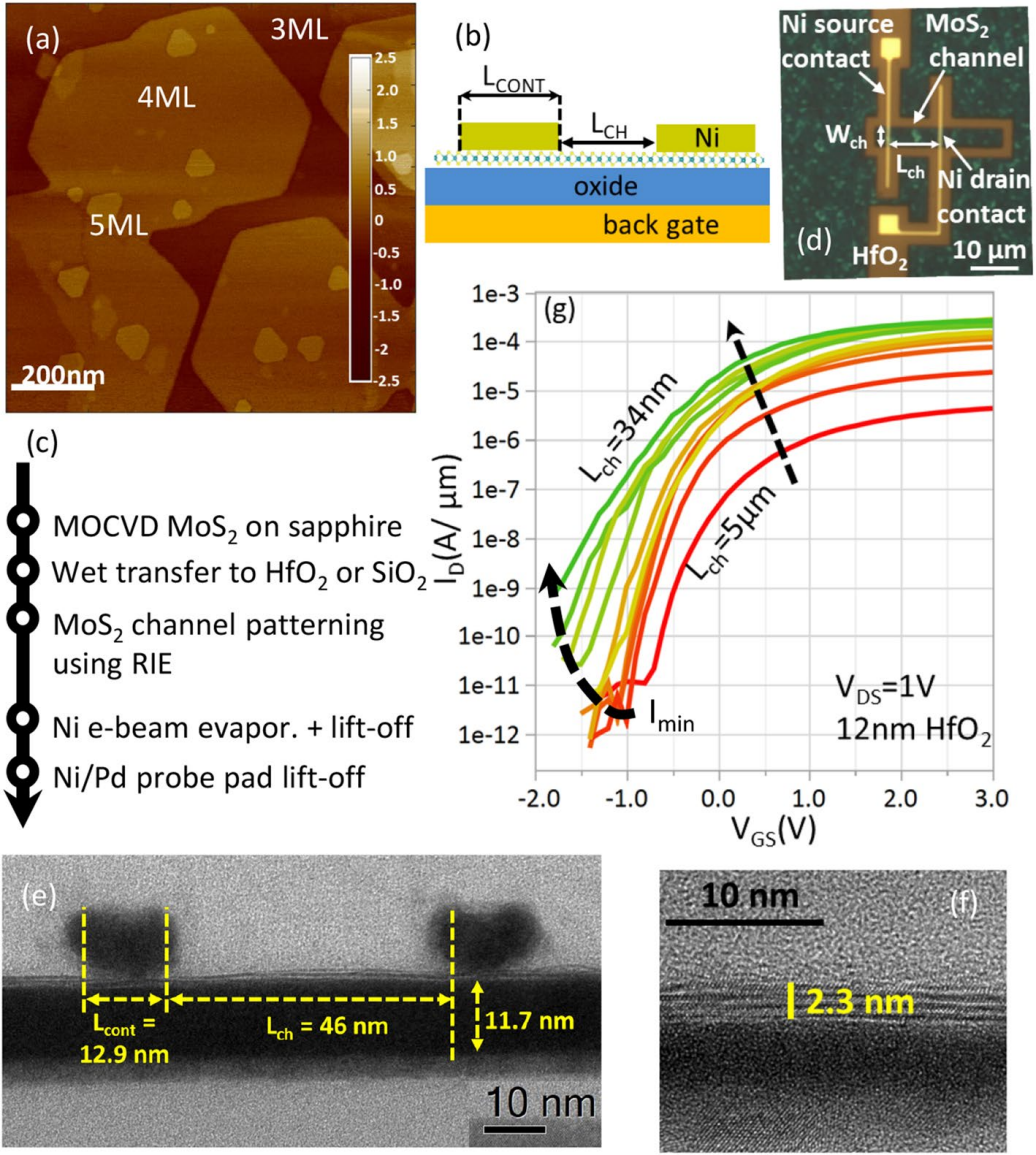
(**a**) Atomic force micrograph of CVD MoS_2_ on sapphire template shows a closed 3ML layer with islands of 4 and 5 monolayers distributed randomly. (**b**) Device schematic with global back-gate and top source/drain contacts. (**c**) Fabrication flow for the back-gated devices. (**d**) Optical micrograph showing the patterned MoS_2_ channel with 10 nm thick Ni contacts. (**e**) Cross-TEM shows a fabricated device with L_cont_ = 13 nm and L_ch_ = 46 nm on 12 nm HfO_2_. (**f**) Zoom-in of the channel region for another device showing 3 monolayer MoS_2_ on nominal 4 nm HfO_2_ (**g**) Transfer characteristics at a fixed V_DS_ = 1 V. Maximum drive current at V_GS_ = 3 V scales with L_ch_ saturating for short-channel devices. The plot shows L_ch_ = 34 nm, 44 nm, 50 nm, 70 nm, 100 nm, 200 nm, 300 nm, 500 nm, 1000 nm, 5000 nm.

We employ large area MoS_2_ grown on a 2″ c-plane sapphire template by metal–organic chemical vapor deposition (MOCVD) process using molybdenum hexacarbonyl and dihydrogen sulfide as the precursors. Atomic force microscopy (AFM) shows the MoS_2_ is composed of 3 monolayers (ML) fully closed and continuous film, with nucleation of 4 ML and 5 ML island regions (Fig. 1a). The average thickness is 3.6 ML, measured using Rutherford backscattering spectrometry (RBS). The device schematic is illustrated in Fig. 1b and details of the fabrication process (Fig. 1c) are discussed in the Methods section. Three different gate-oxides; (1) 50 nm SiO_2_, (2) 12 nm HfO_2_, and (3) 4 nm HfO_2_ are used. An optical image after contact deposition is shown in Fig. 1d and cross-section TEM images of the final fabricated device are shown in Fig. 1e, f.

Direct current measurements are performed in N_2_ ambient to avoid any impact of ambient humidity. A total of 1300 devices with varying L_ch_ (30 nm to 5 μm), L_cont_ (13 nm to 500 nm) and W_ch_ (200 nm to 10 μm) are measured at two different drain-source bias (V_DS_ = 0.05 V, 1 V). Back-gate leakage is low and below the tool noise range (< 1 pA) for the 50 nm SiO_2_ and 12 nm HfO_2_. Devices with 4 nm HfO_2_ have higher gate-drain leakage at V_DS_ = 1 V due to large contact pads. Therefore, the source current (I_S_), instead of the drain current (I_D_), is used in their analysis. Channel edge effects are negligible, as confirmed by the constant on-state current density for several W_ch_ (Fig S1). Devices with short L_ch_, wide W_ch_ and therefore high absolute current, show a large parasitic voltage drop over the source-drain metal probes, and are therefore omitted from the analysis. The threshold voltage of the FETs for V_DS_ = 0.05 V, 1 V is obtained by both the linear extrapolation from peak-transconductance (V_T,LE_) and constant-current method (V_T,CC_ extracted at I_D_ = 10 nA * W_ch_/L_ch_). SS is reported either as SS_min_, which is the minimum value across the entire swing, or as SS_CC_, extracted at a current level of I_D_ = 1 nA * W_ch_/L_ch_ for the stated V_DS_ bias.

### Scaling of on- and off- state currents

**Figure 2:**
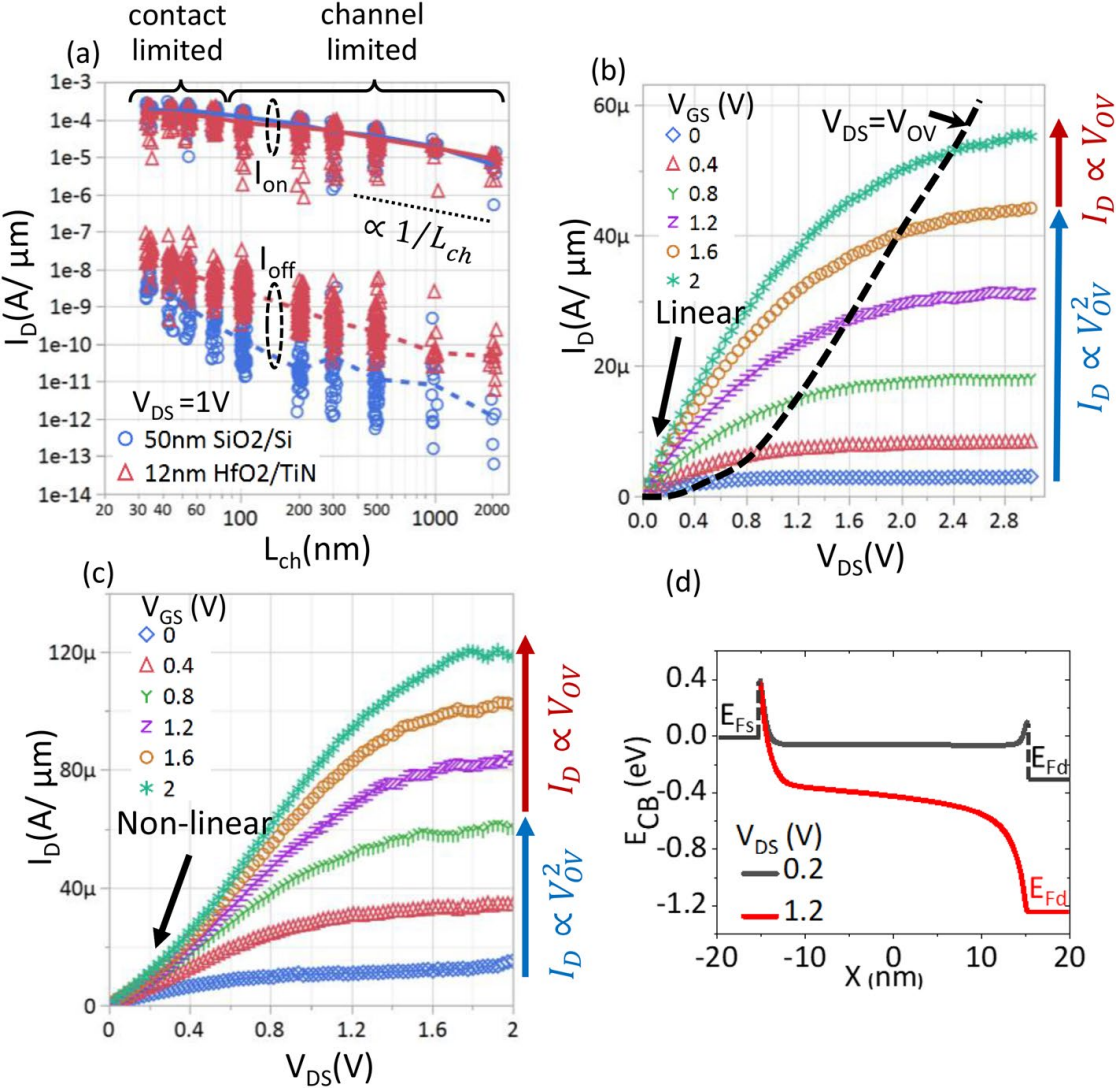
(**a**) Scatter plot (with median line) showing I_on_ extracted at n_s_ = 1e13 cm^−2^ and I_off_ at a fixed displacement field of 0.4 V/nm below V_T,CC._ for V_DS_ = 1 V. I_on_ for the 50 nm SiO_2_ and 12 nm HfO_2_ devices overlap indicating no impact on low-field mobility and contact barrier. I_on_ roughly scales as 1/L_ch_ for L_ch_ > 500 nm and saturates for L_ch_ < 50 nm. I_off_ is higher for HfO_2_ compared to SiO_2_. (**b**) I_D_-V_DS_ for L_ch_ = 500 nm shows linear triode regime and saturation at high V_DS_. The dashed line follows the current at V_DS_ = V_OV._ While the onset of saturation follows the V_OV_ at low V_GS_, it saturates at V_DS_ = 2.4 V for high V_GS_. The saturation current roughly scales V_OV_^2^ and V_OV_^1^, at low and high V_GS_, respectively. (**c**) I_D_-V_DS_ for L_ch_ = 30 nm shows non-linear triode regime due to Schottky contacts and saturation at high V_DS_. Saturation current follows a similar trend as L_ch_ = 500 nm but V_DS_ at onset of velocity saturation is reduced to 1.4 V. (**d**) Conduction band profile for L_ch_ = 30 nm device with Schottky contacts shown for low and high V_DS_. The Fermi-level at the source and drain are indicated by E_FS_ and E_FD_, respectively. The Schottky barrier is shown as the abrupt potential change at the contact-channel interface. At low V_DS,_ I_DS_ is determined by Schottky contacts. At high V_DS_, though the potential drops significantly at the source contact, velocity saturation or pinch-off near the drain determines the I_D_ characteristics.

From the representative transfer characteristics in Fig. 1g, we observe that the off-state current significantly increases as L_ch_ is scaled, as a result of a loss of gate control. Accordingly, we extract the minimum current in the entire back gate sweep (I_min_), and we observe that it is the same for both oxides and lower than the noise floor of the tool (< 1 pA). However, when comparing the I_off_ in the scatterplot Fig. 2a, which is extracted at a fixed displacement field of 0.4 V/nm below V_T,CC_ (i.e.,$|V_{{GS}}  - V_{{T,CC}} |/CET = 0.4\;{\text{V}}/{\text{nm}}$), we note that the HfO_2_ sample exhibits higher I_off_ compared to the SiO_2_ sample. This suggests that the subthreshold swing is limited by the high interface trap density (see *Section D*). We also note that for both oxides, I_off_ degrades with smaller L_ch_. This is mainly due to SS degradation observed for short L_ch_ devices, and will be further discussed in *Section E*.

Next, we evaluate the I_on_ at a fixed charge density (n_s_) of 10^13^ cm^−2^ and do not observe any difference between the 50 nm SiO_2_ and 12 nm HfO_2_ samples (Fig. 2a). This indicates that the carrier transport in the MoS_2_ channel is predominantly limited by charged impurities^14^ in the MoS_2_ or at the interfaces, and not by remote phonons^15^ in the gate oxide.

**Figure 3:**
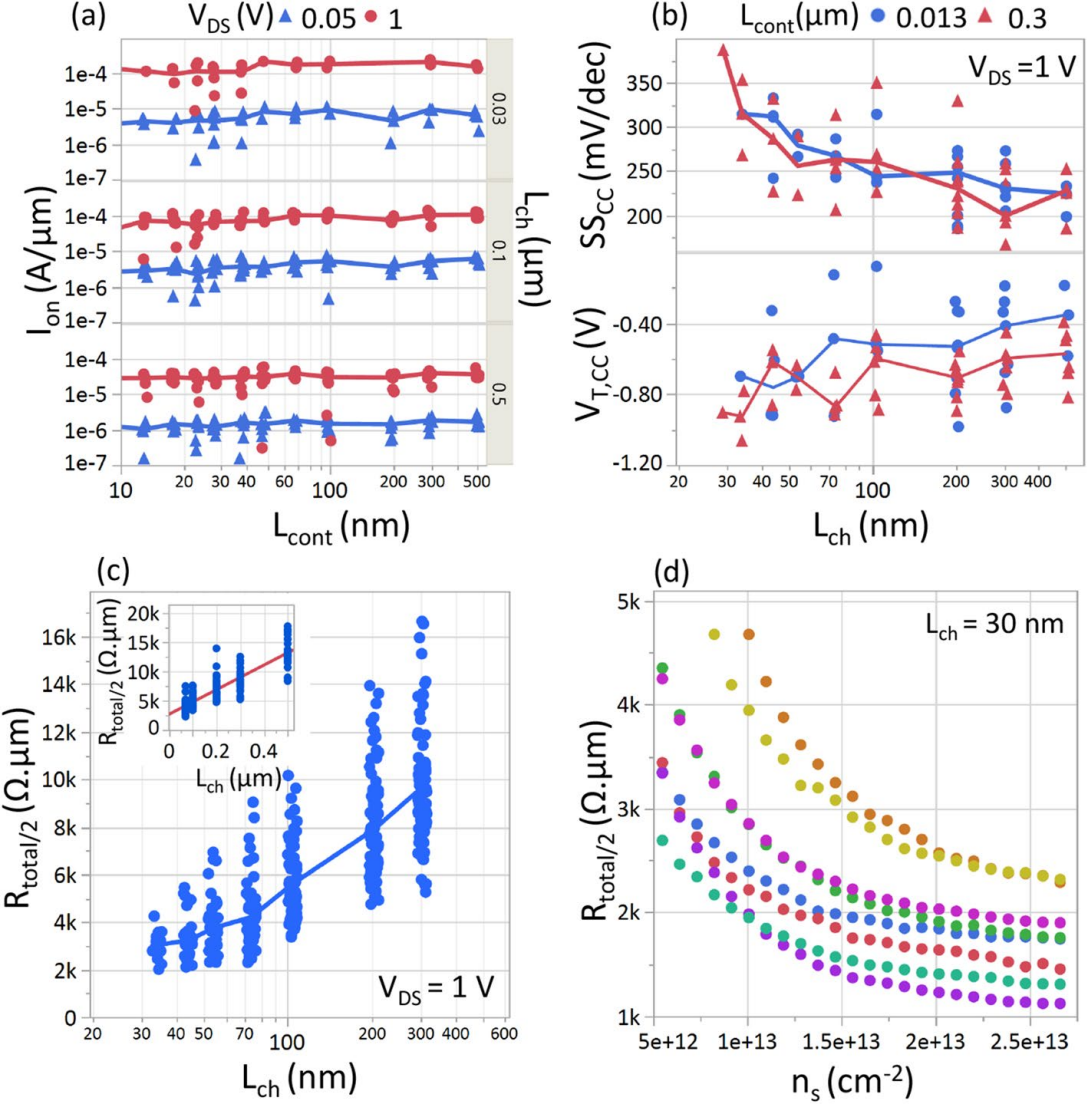
Scatter plot (with median line) of (**a**) I_on_ (at n_s_ = 1e13 cm^−2^) versus L_cont_ for L_ch_ = 30 nm (contact-limited), 100 nm (intermediate regime), and 500 nm (mobility limited). No dependence on L_cont_ down to 13 nm indicates carrier injection from the edge of the metal directly into the MoS_2_ channel with L_T_ < 13 nm. (**b**) SS_CC_ and V_T,CC_ versus L_ch_ for L_cont_ = 13 nm and 300 nm. No systematic deviation with L_cont_ indicates identical electrostatics in both cases. (**c**) R_total/2_ (at n_s_ = 1e13 cm^−2^) versus L_ch_ show saturation below L_ch_ = 50 nm due to contact resistance. Upper limit for R_C_ is obtained as median R_total/2_ for L_ch_ = 30 nm. Median R_C_ values of 3 kΩ.μm with best performers at 2 kΩ.μm are obtained. (inset) TLM fit of R_total/2_ (at n_s_ = 1e13cm^−2^) versus L_ch_ gives R_c_ = 2.7 kΩ.μm and field-effect mobility = 15 cm^−2^/V.s (**d**) R_total/2_ versus n_s_ for L_ch_ = 30 nm at V_DS_ = 1 V of 8 devices. R_C_ significantly reduces at n_s_ = 2e13 cm^−2^ due to better carrier injection into the accumulated channel.

For the I_on_, two distinct channel length scaling regimes can be identified in Fig. 2a. In the long-channel limit (~ L_ch_ > 500 nm), the I_on_ increases roughly proportional to 1/L_ch_ and the device operates in the triode region (illustrated in Fig. 2b for the 12 nm HfO_2_ sample and L_ch_ = 500 nm) i.e. gate-overdrive V_OV_ (= V_GS_ − V_T_) > V_DS_ for both oxides. The drain current also exhibits strongly linear dependence with V_DS_ in the triode region (Fig. 2b), suggesting that the channel resistance is dominant for this L_ch_ and beyond. We also extract a low-field-effect mobility of ~ 15 cm^−2^/V.s (inset of Fig. 3c) using the transfer length method (TLM) for both the samples with 12 nm HfO_2_ and 50 nm SiO_2_. At higher lateral electric field (higher V_DS_), I_D_ saturates (Fig. 2b), and the saturation current scales quadratically with V_OV_ (here V_T,CC_ = −0.4 V) due to channel pinch-off near the drain. However, for the highest V_OV_ (~ 2 to 2.4 V), the saturation current scales roughly linear with V_OV_, indicating that it is limited by saturation of drift velocity at high lateral-field^16^ (F_LATERAL_ > 5 V/μm).

In the short-channel limit (~ L_ch_ < 50 nm), the dependence of I_on_ on L_ch_ saturates (Fig. 2a). Accordingly, in the output characteristics for L_ch_ = 30 nm (Fig. 2c), we make two observations; (1) super-linear I_D_ for V_DS_ < 0.4 V and (2) saturation of I_D_ for V_DS_ > 1.4 V. The distinct super-linear dependence of I_D_ with V_DS_ (Fig. 2c) suggests that the Schottky contacts at the metal-MoS_2_ interface limit the current even though the bias conditions (V_OV_ > V_DS,_ here V_T,CC_ = −0.3 V) ensure that the channel is continuously accumulated with electrons. At higher V_DS_, I_D_ saturates similarly to the L_ch_ = 500 nm device. The current at the onset of saturation is roughly proportional to V_OV_^1.5–1.7^ and V_OV_^0.8–0.9^ for low and high V_OV_, respectively, closely following the long-channel characteristics. This indicates that while contact resistance dominates at low V_DS_, velocity saturation or pinch-off near the drain determines the current at high V_DS_.

We can further understand both these observations from the simulated conduction band profile of L_ch_ = 30 nm device (Fig. 2d) for low and high V_DS._ In the linear regime (V_DS_ = 0.2 V and V_OV_ > V_DS_), the drain-source potential is predominantly dropped across the reverse-biased source and forward-biased drain Schottky contacts. With increasing V_DS_ (higher lateral field), the transmission probability across the Schottky contacts increases rapidly, especially across the reverse-biased source, giving rise to the super-linear dependence of I_D_ with V_DS_. At even higher V_DS_ (V_DS_ = 1.2 V), the electric field in the channel near the drain is large enough to cause either pinch-off at low V_OV_ or saturation of the carrier drift velocity at high V_OV_. Then, this results in saturation of the current.

### Contact length scaling

Figure 3a shows that I_on_ (@ n_s_ = 10^13^ cm^−2^) does not degrade as L_cont_ is scaled down to 13 nm. This agrees with TCAD simulations^10,17,18^ that predict contact edge injection of carriers for 1–3 layers of MoS_2_ channel. This observation holds true for three different L_ch_ (30 nm, 100 nm, 500 nm) over a wide range of L_cont_ (500 nm to 13 nm) and for varying lateral field (V_DS_ = 0.05 V, 1 V). In all three cases, as predicted, we do not observe any systematic degradation of I_on_ by scaling down L_cont_ from 500 to 13 nm. Even for the shortest L_ch_ = 30 nm, where the channel resistance is negligible and the device is Schottky contact limited (I_D_-V_DS_ is super-linear at low V_DS_ in Fig. 2c), the contact resistance is independent of L_cont_. Moreover, the electrostatic properties of the device are also unaffected by scaling down L_cont_ as can be seen in Fig. 3b from the trend of SS_CC_ and V_T,CC_ (@V_DS_ = 1 V) with L_ch_ for two extreme contact lengths. The SS degradation and V_T_ roll-off with shorter L_ch_ are independent of the contact length. The insensitivity to L_cont_ scaling also holds for other gate-oxides and charge densities (plots not shown). In summary, for 3 ML MoS_2_, the active region of MoS_2_ under the metal contact where most of the electrons get injected (called the transfer length L_T_) is at least below 13 nm.

These results agree very well with our previous TCAD simulations with overlapping back-gate. For thin MoS_2_ (1–3 ML), these predict L_T_ smaller than the minimum simulated L_cont_ of 2 nm (Fig S2). This is caused by the Schottky barrier (SB) at metal-MoS_2_ interface, which depletes the MoS_2_ underneath even at a high gate-field and prevents vertical electron injection. Therefore, injection is only allowed from the edge of the metal contact directly into the carrier-rich channel, which is also predicted in other work^18,19^. For thicker MoS_2_ (more than 5 ML), the MoS_2_ region underneath the contact is no longer depleted close to the oxide interface, and a longer section of the contact contributes to carrier injection^20–22^. In a top-gate-only configuration, the absence of gate field under the contact would cause the vertical injection to become even more ineffective for both thick and thin MoS_2_ channels. As a result, the contact length can also be downscaled for top-gated devices without any performance penalty (Fig S2). Moreover, reduction of contact barrier or MoS_2_ sheet resistance under the contact does not increase the L_T_ for 1–3 ML MoS_2_ as the oblique trajectory still provides the least resistive path for carrier injection (Fig S2). However, such improvements could increase L_T_ for thicker MoS_2_ where a substantial carrier injection happens under the contact^19^.

In other work^21,23–25^, transfer lengths of 80 nm to 630 nm have been calculated using the transfer length method (x-axis intercept), but those values are in contradiction with our results. As argued elsewhere^26^, this method should not be used for thin TMD layers and Schottky contacts. The Schottky barrier fully depletes the TMD below, therefore the sheet resistance below the contact and in the channel are not the same, which is a requirement of the transfer length method. However, the transfer length method can still be reliably used for mobility calculation, because it does not have this requirement of identical TMD sheet resistance in the channel and below the metal.

### Contact resistance extraction

As we found in *Section A* that devices become more contact dominated as L_ch_ is scaled, we now take a closer look at the value of the contact resistance. We extract the contact resistance (R_c_) directly as half of the total device resistance (R_tot_/2) for devices with the shortest L_ch_ = 30 nm, without any need for extrapolation like in the TLM method. By considering R_c_ ~ R_tot_/2, an upper limit is obtained for R_c_, as it assumes negligible channel resistance. Figure 3c shows a plot of R_tot_/2 at a charge density of 10^13^ cm^−2^ vs L_ch_. For L_ch_ < 50 nm, the R_tot_/2 saturates, and we obtain a median Nickel-MoS_2_ R_c_ ~ 3 kΩ.μm (at n_s_ = 10^13^ cm^−2^)_,_ which is in good agreement with R_c_ extracted using TLM (inset of Fig. 3c). Our R_C_ values are comparable to the state-of-the-art devices which have been demonstrated with Au^20^ or Indium^27^ contact metals. For increased V_OV_, the contact resistance further drops due to better carrier injection into the accumulated channel, and we obtain R_c_ ~ 1.2–2 kΩ.μm @ n_s_ = 2 × 10^13^ cm^−2^ (Fig. 3d). For even higher carrier densities (compare n_s_ = 2 × 10^13^ cm^−2^ to 2.7 × 10^13^ cm^−2^), R_c_ no longer improves significantly. Significant device-to-device variation in contact resistance is observed, possible due to polymer residues between the contact metal and the MoS_2_, which were not completely removed after the transfer and contact lithography steps of the fabrication flow.

### Long channel electrostatics and D_it_ extraction

**Figure 4:**
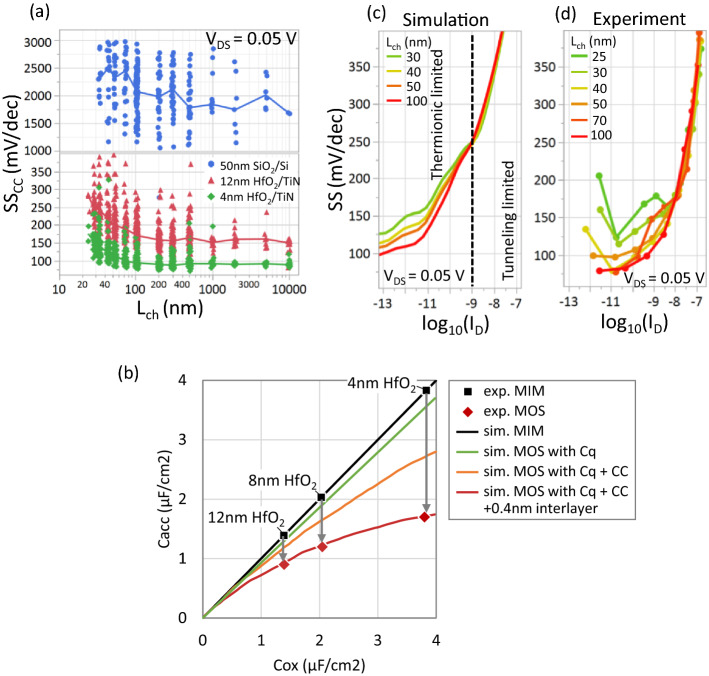
(**a**) Scatter plot (with median line) of SS_CC_ versus L_ch_ for the three different oxides. While SS improves with lower EOT, the degradation and scatter for short channel devices are attributed to electrostatic potential fluctuations caused by non-uniform thickness of MoS_2_ in the contact and channel regions. (**b**) Experimentally measured maximum accumulation capacitance from MOScap (C_acc_) versus MIMcap capacitance (C_ox_). Systematically, the C_acc_ is lower than C_ox_ corresponding to an additional 1 nm CET over the measured EOT. Simulations show this is caused by the quantum capacitance Cq (MoS_2_ having lower DOS than metal), the impact of the charge centroid (CC) further away in MOS than MIM, and additionally due to 0.4 nm of water or carbon residues stuck at the HfO_2_/MoS_2_ interface during transfer. Qualitative comparison between (**c**) simulated and (**d**) experimental SS versus log (I_D_) for different L_ch._ The simulated SS is for a uniform 3 monolayers MoS_2_ with SBH = 0.45 eV. Two transport regimes at the contacts– thermionic emission and tunneling through the SB are identified. In the thermionic regime, the relative increase of field in the channel from the source/drain Schottky contacts degrades gate control for short L_ch_ devices. In the tunneling regime, the nearly equal tunneling lengths for the different L_ch_ results in a similar but degraded SS compared to the thermionic regime.

Figure 4a shows that the subthreshold swing SS_CC_ obtained at V_DS_ = 0.05 V for different L_ch,_ improves with thinner back-gate oxide due to better gate control of the charge in the channel. Consequently, we achieve the best subthreshold swing for the devices on 4 nm HfO_2_ substrate (Fig S3) with median SS_min_ = 90 mV/dec and 110 mV/dec (at V_DS_ = 0.05 V) for L_ch_ = 50 nm and 30 nm, respectively.

In the long-channel limit i.e., L_ch_ > 1 μm, SS_CC_ saturates to a constant median value of 80 mV/dec, 150 mV/dec, 1800 mV/dec for 4 nm HfO_2_, 12 nm HfO_2_, and 50 nm SiO_2_ respectively. This is determined by the charging of MoS_2_/oxide interface and channel defects (60° grain boundaries^28^, and point defects^29^), for which we calculate a trap density (D_it,min_) of 4.5–7 × 10^12^ cm^−2^ eV^−1^ from SS_min_. This range of D_it_ value is roughly similar across the different dielectrics. We also confirm this D_it_ value using multi-frequency C-V measurements of TiN/HfO_2_/MoS_2_ MOScap^30^, where we obtain an acceptor-type trap density of 3.2–6 × 10^12^ cm^−2^ eV^−1^ with energy levels near the midgap.

From C-V measurements, we find that the MOS capacitance is systematically lower than the target oxide capacitance due to exposure to water and/or atmospheric carbon during the wet transfer process from the sapphire template to the target substrates. Figure 4b shows how the maximum accumulation capacitance (C_acc_) measured from TiN/HfO_2_/MoS_2_ MOScap (shown as the red diamonds) is lower than the value of C_ox_ measured from TiN/HfO_2_/TiN MIMcap (without MoS_2_, shown as the black line). Equivalently, the capacitance equivalent oxide thickness (CET) values for MOScap (1.9 nm, 2.7 nm, and 3.8 nm) are systematically 1 nm higher than the EOT values of the MIMcaps (0.9 nm, 1.7 nm, and 2.6 nm). We calculate that the effect of quantum capacitance due to the limited density of states in MoS_2_, and the effect of charge centroid being a few angstrom away from the interface, are insufficient to account for this 1 nm difference. As the MIMcaps are not exposed to water or polymer during the fabrication, Fig. 4b shows the difference between the CET and EOT values can be explained by a 0.4 nm thick layer of water or hydrocarbons adsorbed from the ambient, or a combination thereof. In the future, we expect dry transfer in a controlled ambient will lower the CET, closer to the nominal EOT.

### Short channel electrostatic degradation and variability

In the short-channel limit, i.e., L_ch_ < 100 nm, Fig. 4a shows a degradation of median SS_CC_ but also increased scatter (SS_CC_ at V_DS_ = 1 V in Fig S4). A similar trend is also seen for SS_min_ (Fig S3). We hypothesize that the increased median and scatter could both be caused by the Schottky contacts, where the median SS degradation with shorter L_ch_ is related to the relative increase of depletion regions from the Schottky contacts, while the scatter could be due to the variation in Schottky barrier height^31^ (SBH) induced by the non-uniform thickness of the MoS_2_, seen in the AFM image in Fig. 1a.

We first verify the hypothesis of degraded median SS for shorter L_ch_ by comparing representative experimental SS versus I_D_ curves to simulations in Fig. 4c. We consider full SS–I_D_ curves instead of extracting SS at a single current level to understand the injection mechanism in a wider operation range. The simulations are performed for a SBH = 0.45 eV and uniform 3 ML MoS_2_ channel. We observe two different regimes for SS for both the simulated and experimental data. In the first low-current regime (I_D_ < 1e−9 A/μm), the current is limited by the thermionic emission of carriers from the metal into the channel. Here, the barrier for electrons consists of the highest position of conduction band edge inside the channel determined by the gate-bias. In this low-current regime, SS is determined by the change in the conduction band edge with gate-bias. As discussed in section D, the lower limit for SS (which corresponds to SS_min_ in Fig S3), is defined by the interface trap density. The degradation of SS_min_ for short-L_ch_ devices is due to the electrostatic potential of the source and drain metallurgical junctions influencing the channel potential and degrading the gate control. This is illustrated in Fig S5 where the conduction band energy is flat over most of the device for L_ch_ = 100 nm, while it is lowered for L_ch_ = 30 nm with the region of maximum barrier reducing to a small portion near the center of the device. Note that this effect is similar to conventional MOSFETs.

The second regime (I_D_ > 1e−9 A/μm) is reached when the conduction band in the channel is lowered further, and carriers can efficiently tunnel through the SB (Fig S6). Here, the thermionic component over the barrier saturates and the tunneling path length determines the current. Because it continuously changes with higher V_GS_, the SS is worse than the first regime. Correspondingly, in the experimental devices, the SS_CC_ extracted at I_D_ > 1e−8 A/μm (for L_ch_ < 100 nm) shows a higher value than SS_min_ and stronger degradation with L_ch_. The SS for a given I_D_ also becomes nearly independent of L_ch_, because the tunneling path length depends only on the gate voltage and the thicknesses and dielectric permittivities of the TMD^32^ and oxide, for the low lateral electric field (V_DS_ = 0.05 V). This is illustrated in Fig S6 where the conduction band energy and tunneling rate are plotted along the edge carrier injection path for L_ch_ = 30 nm and 100 nm, showing no significant difference. With further reduction in SBH, the SS value in the second regime improves, reaching closer to the thermionic limit of the first regime.

**Figure 5:**
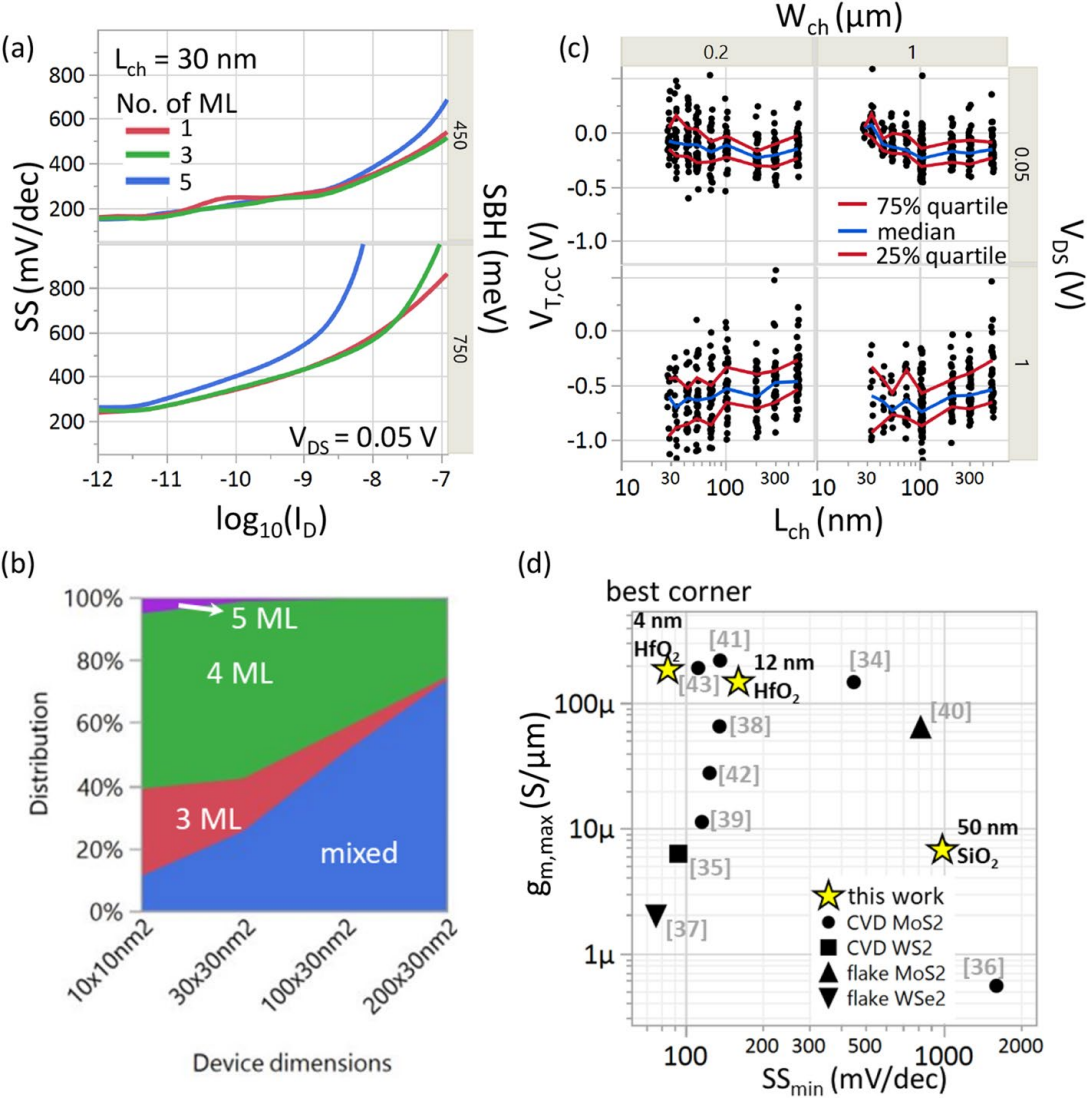
(**a**) Simulated SS versus log (I_D_) for a uniform layer of 1, 3 and 5 monolayers of MoS_2_ for SBH = 0.45 eV and 0.75 eV. For I_D_ > 1e−9 A/μm, tunneling through Schottky barrier determines the SS. Subsequently, a thinner channel results in better gate control, shorter tunneling length and therefore better SS. (**b**) Probability distribution versus device dimensions (L_ch_ x W_ch_). AFM from Fig. 1a was used to compute the probability distribution for fabricating devices on only 3, 4, 5 or a combination of those (mixed). Our experimental devices have a 60–70% probability of being mixed, leading to non-uniform gate control across the channel and contact regions. (**c**) V_T,CC_ versus L_ch_ for two different W_ch_ (200 nm, 1000 nm) and V_DS_ (0.05 V, 1 V). No V_T_ roll-off at V_DS_ = 0.05 V due to excellent gate control over the channel for 12 nm HfO_2_. V_T_ roll-off of about 200 mV for V_DS_ = 1 V due to higher lateral-field at the source contact allowing for more carrier injection. No systematic V_T_ deviation between W_ch_ = 1 μm and 200 nm. (d) Benchmark plot showing g_m,max_ versus SS_min_. All values are at V_DS_ = 1 V except^39^—V_DS_ = 0.1 V^34^,—V_DS_ = 0.5 V^40^,—V_DS_ = 1.2 V^37^,—V_DS_ = 1.5 V. In this work, 4 nm HfO_2_ provides best SS = 86 mV/dec and g_m,max_ = 185 μS/μm.

We study the increased SS scatter for short L_ch_ seen experimentally, using simulations of devices with different uniform MoS_2_ channel thickness and SBH. Figure 5a shows the simulated SS value for two different SBH (0.45 eV, 0.75 eV) and three different uniform thicknesses (1, 3 and 5 layers) of MoS_2_ for L_ch_ = 30 nm. Similar to the above case, we note two different regimes for SS irrespective of the barrier height. For the first regime of low I_D_ (< 1e−8 A/μm for SBH = 0.45 eV and < 1e−11 A/μm for SBH = 0.75 eV), the SS is determined only by thermionic emission over the channel barrier. Therefore, the SS is independent on the channel thickness. However, the SS degrades for SBH = 0.75 eV compared to 0.45 eV, because the higher Schottky barrier field penetrates deeper into the channel. For the second regime of high I_D_ (> 1e−8 A/μm for SBH = 0.45 eV and > 1e−10 A/μm for SBH = 0.75 eV), the SS is dependent on the tunneling length which is sensitive to the thickness of the semiconductor among other parameters^33^. Subsequently, the gate control over the Schottky barrier, and hence the tunneling length, reduces with thicker MoS_2_, resulting in poor SS for the 5 ML MoS_2_ (Fig S7). In agreement with this observation, we also note that the difference in SS between the layers is more pronounced for the higher SBH of 0.75 eV.

In our experiments, we have even more variability due to non-uniform thickness within a single device. Even for the smallest functional device footprint (L_ch_ ~ 30 nm * W_ch_ ~ 200 nm), we always have a high probability (~ 70%) of having a mixed device i.e., regions of 3, 4 and 5 layers of MoS_2_ within the same device. This is illustrated in Fig. 5b where the representative AFM (Fig. 1a) image of the material was used to compute the probability of fabricating devices with different dimensions on only 3 (or) 4 (or) 5 or a mix of those layers. These mixed-thickness devices, together with the associated SBH variations, would result in non-uniform gate control and large scatter in the SS values of experimental devices. Also, note that the grain size and defects in the closed layers (1–3 ML) could additionally impact the device variability.

### Threshold voltage control

We analyse V_T_ control for decreasing channel length, and Fig. 5c shows that there is no significant median V_T_ roll-off at V_DS_ = 0.05 V. With a higher V_DS_ = 1 V, we notice a V_T_ roll-off of about 200 mV from L_ch_ = 500 nm to 30 nm. We attribute this roll-off to the higher lateral electric field across the reverse-biased Schottky contact, because V_DS_ is fixed at 1 V for all L_ch_. This higher electric field allows for increased carrier injection in short channel devices, which lowers V_T_. This roll-off could be mitigated by improving the gate control through gate-oxide scaling, or by reducing the amount of defects at the MoS_2_/oxide interface.

V_T_ control for decreasing channel width is also shown in Fig. 5c, and no systematic impact is seen as W_ch_ is scaled from 1 μm down to 200 nm. However, we note that the narrow devices (W_ch_ = 200 nm) show higher V_T_ variability than wider devices (W_ch_ = 1 μm), especially at V_DS_ = 0.05 V. This increased V_T_ variability could be attributed to the higher probability of finding devices on discrete layers (Fig. 5b) for narrower channel compared to a wider channel where the devices are always mixed. Other sources of variability such as bias-temperature instability, non-uniformity of the MoS_2_ grains etc. could also impact the V_T_ variability and more dedicated experiments are required.

## Benchmark, projection and conclusion

We present a benchmark chart (Fig. 5d) to compare the performance of our devices against flake and CVD 2D material FETs in literature^34–43^. We choose the peak of transconductance (g_m,max_) measured at V_DS_ = 1 V and SS_min_ as the two metrics for comparison, similar to conventional Si transistors. The best corner is on the top-left since low SS_min_ and high g_m,max_ are desired. Our SiO_2_ devices, owing to the thick EOT, provide low transconductance even for the shortest L_ch_ devices. Scaling the EOT (12 nm HfO_2_ and 4 nm HfO_2_) and using an optimized process flow (see Methods), we gain both in transconductance and SS, achieving a R_c_ < 2 kΩ.μm for Ni contact metal and D_it_ < 5 × 10^12^ cm^−2^ for a CET of 1.9 nm. We demonstrate the highest g_m,max_ = 185 μS/μm at V_DS_ = 1 V and a minimum SS of 86 mV/dec for 4 nm HfO_2._ We also achieve I_max_ = 400 μA/μm at V_DS_ = 1 V and V_GS_ = 4 V for our 12 nm HfO_2_ samples (Fig S8).

**Figure 6:**
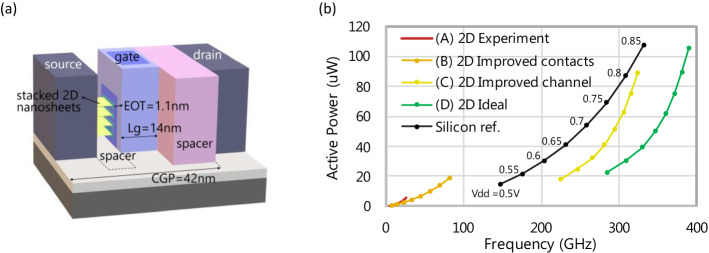
(**a**) Power performance area (PPA) analysis comparing silicon and 2D in the same configuration of 4 stacked nanosheets with gate-all-around. (**b**) The baseline (A) is set with experimental values, R_c_ = 1.5 kΩ.µm (corresponding to Φ_SB_ = 0.45 eV), µ = 15 cm^2^/Vs, D_it_ = 3 × 10^12^ cm^−2^ eV^−1^, t_channel_ = 3 layers. For (B) the contacts are improved with R_c_ ≤ 50 Ω.µm (corresponding to Φ_SB_ = 0.2 eV). For (C) the channel is further improved with µ = 200 cm^2^/Vs, D_it_ = 1 × 10^12^ cm^−2^ eV^−1^, t_channel_ = 1 layer. For (D), more aggressive improvements are done with µ = 450 cm^2^/Vs and no R_c_. For all curves, the area is the same and the bias conditions are such that at V_dd_ = 0.7 V, I_off_ is fixed at 2nA. Methodology from^44^.

Despite the fact our 2D performance is among the best in literature, significant improvements are still needed to make 2D materials competitive with silicon channel devices for high-performance logic applications. Therefore we propose a roadmap using the Power Performance Area (PPA) metric for technology comparison in Fig. 6. 2D-FET and silicon nanosheet technology are compared using an inverter-based ring oscillator circuit, where each device consists of 4 vertically stacked sheets with scaled L_g_ = 14 nm and gate-all-around structure, corresponding to the imec 2 nm node^44^. All devices are retargeted to an I_off_ = 2 nA at V_dd_ = 0.7 V and the inverter-circuit area is kept the same for fairer comparison between technologies. Starting from the baseline case (A) where experimental channel and contact parameters are assumed, the performance strongly improves in (B) when the Schottky barrier height is reduced. In (C), improvements to the 2D channel mobility results in higher ring-oscillator operating frequency compared to silicon, owing to superior electrostatic control of the 2D devices at shorter gate lengths. In (D), the ideal performance is simulated with more aggressively optimized material parameters.

In conclusion, we have scaled down the different device dimensions of CVD-grown MoS_2_ FETs and demonstrated g_m,max_ = 185 μS/μm and SS_min_ = 86 mV/dec which are among the best in literature. Using our large dataset, we systematically identified the key obstacles to be tackled to outperform silicon. First, we showed that scaling L_cont_ for thin MoS_2_ does not impact the short channel performance, which allows for an overall reduction in the device footprint and enables device and circuit level gate optimization^45^. Second, we identified that for L_ch_ < 100 nm, the on-current is currently limited by high Schottky contact resistance (R_c_ = 1–2 kΩ.μm) at low V_DS_, and by a combination of velocity saturation and the Schottky barriers at high V_DS_. Third, we identified that our devices suffer from short channel effects (SS degradation), caused by the Schottky barrier at intermediate current level and the thick CET at low current level. Reducing the CET is therefore crucial to keep optimal electrostatic control of the thin channel. We established that a 0.4 nm layer of water or adsorbed hydrocarbons (or combination thereof) at the HfO_2_/MoS_2_ interface is the root cause of a lower-than-expected CET. This value is consistent across different thicknesses of HfO_2_. Therefore, an optimized transfer process free of water and carbon is needed to enable gate stack scaling below 1 nm, and additionally allow upscaling to 300 mm-wafer processing. Finally, we have demonstrated using a PPA analysis that if the obstacles of Schottky contacts, gate stack scaling and mobility improvement can be tackled, MoS_2_ FETs will significantly outperform silicon GAA FETs at the imec 2 nm node and beyond. Therefore, they are excellent candidates to continue logic scaling.

## Methods

### Device fabrication

For the device design, we use the back-gate configuration with top-contacts (Fig. 1b). The fabrication flow is summarized in Fig. 1c. The MoS_2_ is delaminated from the sapphire growth substrate using water intercalation and transferred to three different target substrates; (1) Si/50 nm SiO_2_ (2) Si/50 nm SiO_2_/5 nm TiN/12 nm HfO_2_, or (3) Si/50 nm SiO_2_/5 nm TiN/4 nm HfO_2_. Before transfer, the target substrates are pre-cleaned using a solvent rinse, followed by an optimized forming gas anneal (FGA) or soft O_2_ plasma, for SiO_2_ and HfO_2_ back-gate oxides, respectively. The active channels are patterned using PMMA mask and e-beam lithography, followed by reactive ion etching (Cl_2_ + O_2_) of MoS_2_. Source and drain contacts of different lengths (L_cont_) with different channel lengths (L_ch_) are subsequently defined on the active channel by another e-beam lithography exposure of ZEP520A-2 resist (ZEON Corp.), e-beam evaporation of 10 nm Ni, and metal lift-off in anisole. We ensure a low vacuum pressure < 10^–6^ Torr while depositing the Ni contact metal. Finally, in a third e-beam lithography step, thicker Ni/Pd contact pads are lifted off.

### TCAD calibration

All simulations^46^ are performed in Sentaurus Synopsys Device. The low-field mobility (μ_eff_) is calibrated from an experimental TLM fit shown in Fig. 3c and implemented under a constant mobility model. An estimate for D_it_ is obtained from multi-frequency CV measurements as discussed in section D. An acceptor trap distribution uniform over the entire bandgap is assumed with D_it_ = 3e12 cm^−2^ eV^−1^. With μ_eff_ and D_it_ fixed by experiments, the Schottky barrier height is fitted to median transfer characteristics of L_ch_ = 30 nm devices which are predominantly contact-limited. For the Schottky injection, the non-local tunneling model based on the Wentzel-Kramers-Brillouin approach is used. All the parameters used in the simulation correspond to their median values.

## Supplementary Information

Supplementary Information

## Data Availability

The data that support the findings of this study are available from the corresponding author upon reasonable request.
