# Supplementary material for: Impact of device scaling on the electrical properties of MoS2 field-effect transistors
Source: Sci Rep. 2021 Mar 23;11:6610. doi: 10.1038/s41598-021-85968-y (PMC7987965; doi:10.1038/s41598-021-85968-y)
Supplement: Supplementary file 1 — Supplementary Information [file 41598_2021_85968_MOESM1_ESM.pdf]

Impact of device scaling on the electrical properties of MoS_2 field-effect transistors

Goutham Arutchelvan[1,2], Quentin Smets[1], Devin Verreck[1], Zubair Ahmed[1], Abhinav Gaur[2], Surajit Sutar[1], Julien Jussot[1], Benjamin Groven[1], Marc Heyns[1,2], Dennis Lin[1], Inge Asselberghs[1] and Iuliana Radu[1]

[1] imec, Leuven, Belgium

[2] KU Leuven, Belgium

A. Impact of channel patterning on device performance

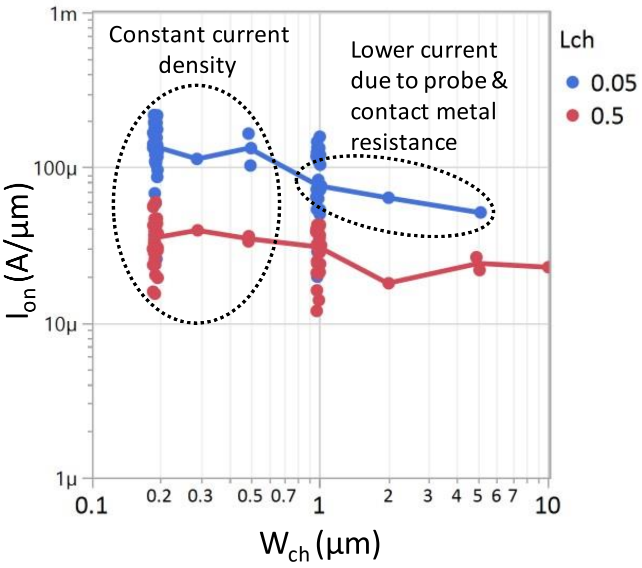

Fig S1. Scatter plot (with median line) of I_{on} (at n_s = 1e13 cm^{-2}) versus W_{ch}. The current reduction for large channel width is due to increased potential drop in the probe metal. The potential drop is more pronounced for short channel devices where the drive current is higher.

B. Simulation to evaluate the impact of contact length scaling

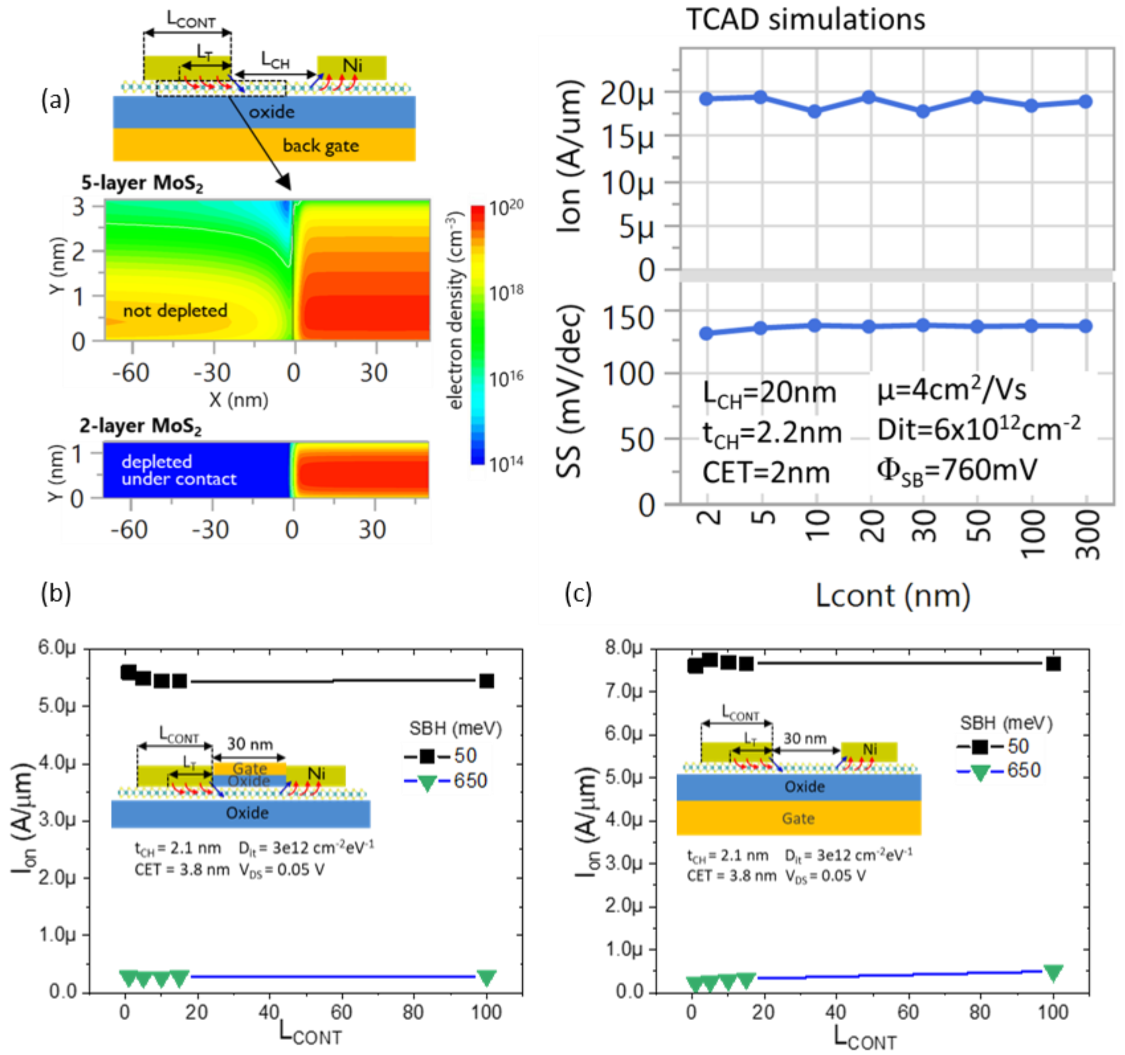

Fig S2. TCAD simulations showing (a) no impact of contact length scaling for 3 monolayers MoS_2 and overlapping back-gate. Compared to a thick (5 monolayers) MoS_2 where the region under the contacts can accumulate carriers at high gate-bias, the 3 monolayers remain depleted for all bias conditions. This results in the contact path (blue arrow in the schematic) as the most dominant carrier injection path. Thus, scaling the contact length has no impact on the I_{on} (at V_{OV} = 0.7V) or the SS of the scaled devices. (b) the contact length can also be scaled for top-gated devices as the absence of gate field under the contact renders the vertical electron injection even more ineffective. Here I_{on} extracted at V_{OV} = 0.7V. (c) for 1-3 ML MoS_2 FETs improving sheet resistance under the contact by reducing the barrier height has no impact on the transfer length. This is mainly due to the exponential increase in thermionic and tunneling current close to the contact edge with decreasing SBH, which has a higher impact than sheet resistance improvement. Thus, the I_{on} (at V_{OV} = 0.7V) is independent of L_{cont} similar to the other cases.

C. Minimum Subthreshold swing

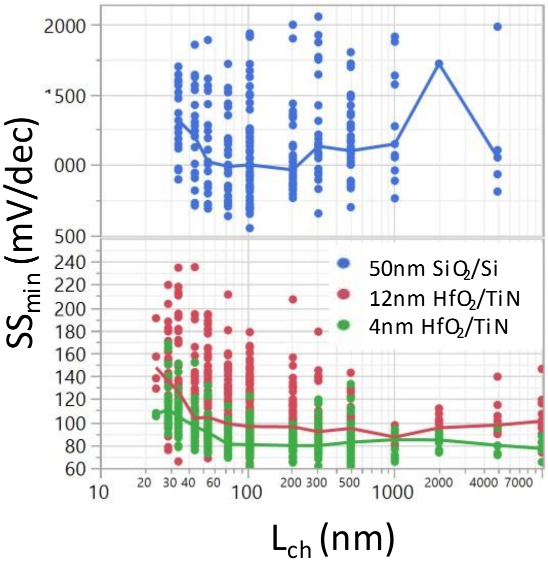

Fig S3. Scatter plot (with median line) of Minimum Substhreshold swing (SS_{min}) versus L_{ch} for V_{DS} = 0.05 V. SS_{min} improves with decrease in EOT. Best values are obtained with 4 nm HfO_2 (CET = 1.9 nm). Median SS_{min} of 110 mV/dec and 90 mV/dec are obtained for L_{ch} = 30 nm and L_{ch} = 50nm, respectively. For L_{ch} > 100 nm, we roughly have a median SS_{min} of 80 mV/dec.

D. Subthreshold swing comparison between V_{DS} = 1 V and 0.05 V

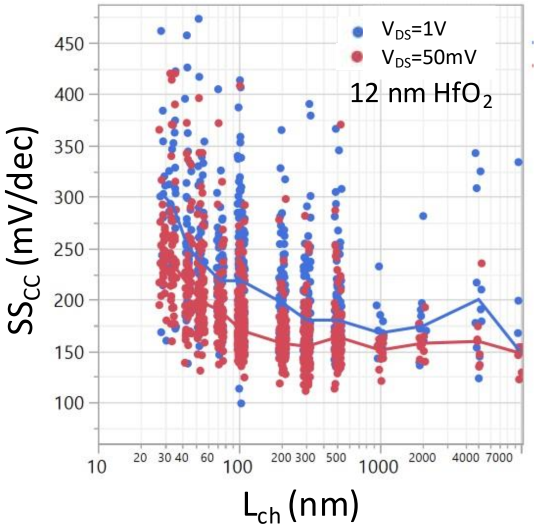

Fig S4. Scatter plot (with median line) of SS_{CC} versus L_{ch} shows a slightly degraded subthreshold swing for V_{DS} = 1 V. The degradation could result from repeated measurement of the device without allowing for relaxation of the interface/oxide traps. SS at V_{DS} = 1 V also shows a stronger degradation upon scaling L_{ch}.

E. Impact of Schottky barrier on device electrostatics at off-state

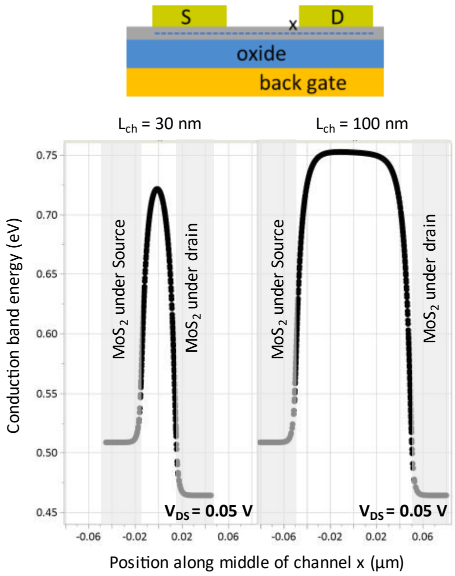

Fig S5. Conduction band energy plotted across the middle of the channel for SBH = 0.45 eV and same gate-bias. The barrier for electrons is reduced for L_{ch} = 30 nm as the source and drain Schottky depletion regions penetrate deeper into the channel reducing the gate control. This leads to higher subthreshold current and poor SS as the L_{ch} is reduced.

F. Impact of channel length on tunneling rate in subthreshold region

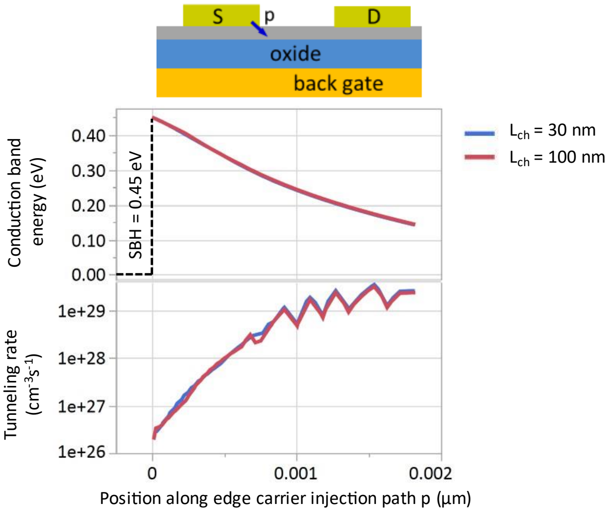

Fig S6. Conduction band edge energy and tunneling rate plotted at a gate-bias corresponding to the tunneling regime of the subthreshold region for V_{DS} = 0.05 V. The tunneling rate is the semiclassical electron generation rate at the end of the tunnel path. The conduction band edge and tunnel rate are plotted along dominant injection path, which is diagonal from the edge of the source metal, as shown in schematic. Beyond the flat-band voltage, carriers can tunnel from the metal into the channel. This makes the SS, in general, worse than the thermionic limit seen in Fig S4. The tunneling rate is slightly higher for L_{ch} = 30 nm due to slightly increased lateral electric field, resulting in a slightly better SS compared to L_{ch} = 100 nm.

G. Impact of channel thickness on tunneling rate in subthreshold region

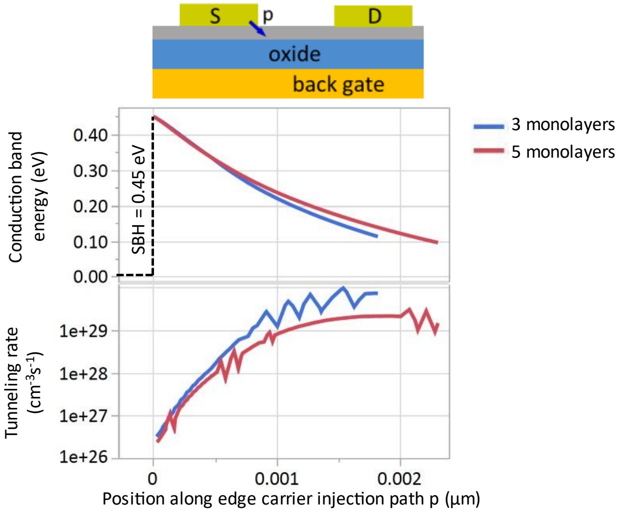

Fig S7. Conduction band energy and tunneling rate plotted at gate-bias corresponding to the tunneling regime of the subthreshold region for V_{DS} = 0.05 V. The quantities are plotted along the contact edge injection path as shown in schematic. The tunneling length for five monolayers MoS_2 is longer leading to a lower tunneling probability. This degrades the SS in the regime beyond flatband voltage and the difference between the layers become more pronounced with increasing Schottky barrier height.

H. I_{max} vs SS_{min} for best performers

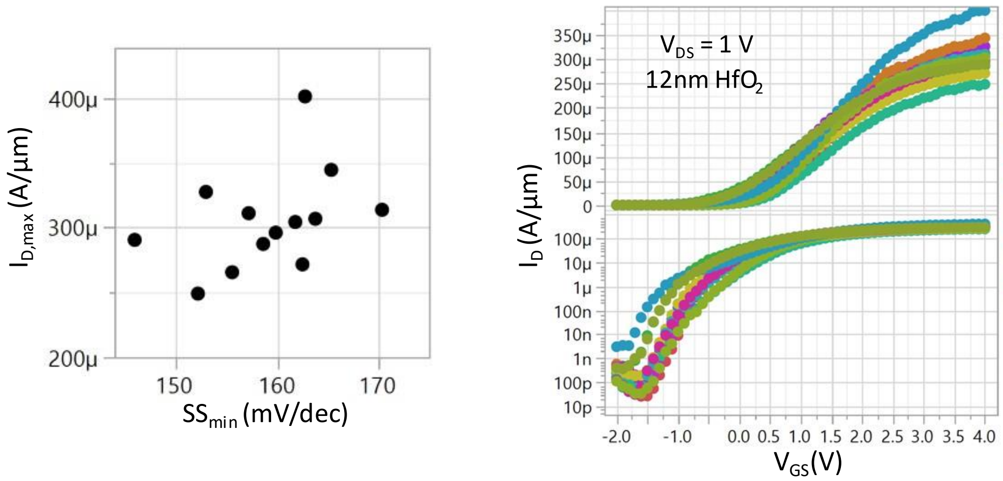

Fig S8. I_{max} versus SS_{min} shown for the best performing devices with 12nm HfO_2. Transfer characteristics of the same devices showing $I_{max}/I_{min} > 10^6$. At high $V_{GS} > 2$ V, the devices are limited by MoS_2-Ni contact and probe metal resistance.
